# Drainage ditches enhance forest succession in a raised bog but do not affect the spatial pattern of tree encroachment

**DOI:** 10.1371/journal.pone.0247760

**Published:** 2021-03-18

**Authors:** Joanna Nowakowska, Anna Gazda, Andrzej Tomski, Jerzy Szwagrzyk

**Affiliations:** 1 Department of Forest Biodiversity, University of Agriculture in Krakow, Krakow, Poland; 2 Institute of Mathematics, University of Silesia in Katowice, Katowice, Poland; Virginia Commonwealth University, UNITED STATES

## Abstract

The study was conducted in the raised bog Kusowo (Baltic region, West Pomerania, Poland). Along a transect line crossing two open mires affected by forest succession we analysed tree age distribution. One of those mires had been drained in the past years and still retained some open ditches, while the other one was located far from the ditches. Every 10 meters along the transect line one tree was drilled at the root collar in order to determine its age. We also conducted phytosociological analyses and short-term water level measurements in the sample plots. We expected faster tree encroachment in the undisturbed part of the open mire. The results showed, that there were no significant differences in water table level and in soil moisture indicator values between the formerly drained and undisturbed open mire. There were also no statistically significant differences in tree encroachment between the disturbed and undisturbed mires. Location and the age distribution of the trees suggest that changes in the tree growth conditions cannot be directly explained by the general decreasing of water level in the bog, although periods following drainage works were associated with more numerous establishment of young trees, in the drained part of the bog as well as in the part not directly affected by drainage ditches.

## Introduction

Peat bogs are among the most important ecosystems for water retention [1], they are able accumulate large amounts of water during heavy rains or snowmelt, and then discharge the water slowly. They also play a large role in carbon storage [2, 3]; peat bogs and other wetlands at the global scale contain more carbon than forests do. However, two types of communities comprising spatial-ecological system of raised bogs: open mires and bog woodland are among the most endangered habitats in Europe [4]. The processes of shrinking of lakes and associated peat bog development have been going on gradually throughout the Holocene [5], but in present times it has been intensified by global changes, especially climate warming [6] and the eutrophication effects caused by increased availability of nitrogen [7]. There is an urgent need to understand the processes shaping their structure and species composition to maintain peat bogs in the landscapes strongly affected by global changes and by various forms of human management, like peat extraction, decreased and unstable water resources, pollution from agriculture [8, 9]. Among many threats for raised bog conservation are the long-term effects of former drainage; they cause the increase in tree cover, that in turn affects the water balance of the bog by increasing the water transpiration [10–13]. Among tree species encroaching onto open mires are: Norway spruce *Picea abies*, Downy birch *Betula pubescens*; however, a major role is usually played by Scots pine *Pinus sylvestris*. Scots pine is the most widespread tree species in the eastern Europe, inhabiting a wide range of habitats, from relatively poor and dry soils developed from post-glacial sand deposits to extremely wet habitat on the margins of peat bogs. It is also a light demanding tree species, and can reach very old age, especially when growing slowly in harsh environmental conditions [14]. Pine encroaches onto open mires when site conditions changes to more dry [15–17]. Sometimes, due to the natural processes water level increases and the trees die back [18]. In natural conditions, open mires are gradually changing into bog woodland in the process of succession [19], and the rate of that process depends on the relationship between precipitation and transpiration [11, 20, 21]. In hot and dry years, the water table goes down and new tree seedlings are able to establish in open mires. In wet and cool years, the water table level increases and some of the already established seedlings can die. The seeds of Scots pine are able to germinate in the *Sphagnum* mosses dominated environment. Once the Scots pine gets established in peat bogs, it has been gradually changing the environmental conditions and vegetation structure in the close neighbourhood. Scots pine is capable of impeding peat accumulation in a significant way [22]. However, the changed conditions in the vicinity of already established pine trees are less conducive for germination and establishment of pine seedlings. Thus there is no positive feedback of pine establishment upon the further pine recruitment, and that allows to maintain a kind of balance between trees and open mire habitats in raised peat bogs, as long as the water table level is not significantly and permanently lowered [22].

Drainage breaks this natural cycle of tree establishment and tree dieback allows Scots pine to persist in open mires and eventually turn it into a pine bog forest. Intensified tree encroachment as a result of drainage was reported in numerous studies [11, 19, 23–28]. Increase in the tree cover as a result of long-lasting legacy of former drainage human-related disturbances, causes decreasing and withdrawal of the major functions of raised bog ecosystems regarding a water retention and carbon sequestration balance on the landscape level. This happens mainly due to the fact that the extraction of water by tree roots increases the water losses from the peat. Numerous research reported the stimulating role of drainage ditches to bog overgrowing, according to the lower water level near the ditches edges [29–31]. In the experimental studies, ditch sides were considered as more appropriate sites for tree growth than adjacent sites due to the shift from anoxic to oxic conditions in the root zone caused by lowered water table level [30]. The second factor differentiating tree encroachment is the distance to the nearest mature trees [32].

The aim of this study was to analyse the spatial dynamics of the system of open mires (*7110 Natura 2000 site) and bog woodlands (*91D02 Natura 2000 site). Because of the draining of the mire conducted in the past, the spatial relationship between these two communities had been probably changed in a substantial way.

Our first hypothesis was that the tree encroachment onto open mires is related to the distance to the nearest ditch; we expected Scots pines to get established in higher numbers drained part of the peat bog, where the lowered water table should have produced conditions more conducive to tree growth and survival.

Our second hypothesis was that the intensity of tree encroachment was greatest just after building the ditches, and then it has gradually slowed down. The ditches have not been properly maintained, and since the establishment of the reserve they were abandoned, and nowadays they are not visible in aerial photographs. Thus the role of drainage ditches in promoting tree establishment should have been more pronounced in the past, when their effect on water table level was probably much stronger than in recent times.

## Materials and methods

### Study area

The study was conducted on the Baltic bog located in the west Pomerania (north-western Poland), in the Kusowo Bog Nature Reserve (53°49’0.00"N, 16°34’60.00"E) covering the area of 326 ha. This peat bog has been considered as one of the best preserved raised peat bogs in Poland. Research area comprises several mires gradually overgrown by the Scots pine (*P*. *sylvestris*) from the edges towards the central, open mire. About two-third of the reserve is now covered with bog forests *Vaccinio uliginosi Pinetum sylvestris* and *V*. *uliginosi-B*. *pubescentis*, and one third is an open mire, mostly the *Sphagnetum magellanici* and the *Eriophorum vaginatum-Sphagnum recurvum* community. There are also small patches of beech forests on the mineral deposits on the margin of the reserve.

The mean annual temperature is 7.5°C, the annual sum of precipitation is 610 mm, out of that 400 mm during the growing season, that lasts approximately 210 days, from the beginning of April until the end of October. The depth of the peat reaches up to 12 m. The elevation of the entire reserve ranges between 145 and 159 m above sea level, and the denivelation within the peat bog does not exceed 2.5 m. The area under study was about 150 ha, comprising the least disturbed northern part of the Kusowo bog reserve. There are 279 species of vascular plants, including rare ones like *Baeothyron cespitosum*, *Carex limosa*, *Drosera anglica*, and *Drosera rotundifolia*.

The drainage operations were carried out during 1930’s and 1970’s, but the older documents and maps suggest that the bog had been drained also in the 20th century, when the exploitation of peat started in what is now a southern part of the Kusowo reserve. No peat exploitation was ever done in the northern part of the reserve, where we conducted our study [33].

### Data collection

Three transects were established running in the east-west direction through the Kusowo Bog Nature Reserve. Along the transect lines 70 sample plots were placed in the middle of each distinct vegetation patch crossed by a transect line in these sample plots phytosociological relevés according to the Braun-Blanquet method [34] were made to determine the plant community type and the water table depth was monitored using 1 m long piezometers throughout one year, once per two weeks during the growing season and once per month in winter.

The middle one of the three transect lines, with a length of 1122 m was chosen for conducting the analyses of the age structure of trees. The transect crossed two mires gradually overgrown by the pine trees: one with the presence of the open ditches (at present overgrown by bog vegetation; no longer active; so called ’disturbed mire’) and another mire without any presence of the ditches confirmed by available cartographic materials (so called: ’undisturbed mire’). The lengths of transects in the disturbed mire and undisturbed mire amounted to 331 m and 277 m, respectively.

Along the transect line every 10 m at least one nearest tree was selected for age determination. The height of each investigated tree was measured (S1 Table). In case, where there were several trees near the sample point, only the largest and the smallest tree were measured. Trees that were at least 30 mm thick at the mire surface were drilled with the Pressler increment borer as close to the ground as possible, in order to determine the number of annual rings. The number of rings was considered to represent tree age at the height of coring. In order to determine the actual age of each tree, an estimation of the number of years necessary to reach the height of coring was made. This estimation was based on a sample of small pine trees (max. 20 mm in diameter at the surface of the mire) collected in adjacent open mire. These small trees were extracted from the ground and analysed in the laboratory for determination of number of years necessary to grow from the root collar to the height of coring [35, 36].

For conducting the field work in the nature reserve we obtained a permit number WOPN.6205.55.2.2011.JS, issued on December 2, 2011 by the Director of the Regional Agency of Nature Conservation in Szczecin.

### Statistical analyses

On the basis of the phytosociological relevés we calculated the Ellenberg’s indicator values [37] for each sample plot. These indicators are estimates of environmental variables Soil Moisture value F, Nitrogen value N and Soil Reaction value R, calculated as the mean of the indicator values for the list of plant species present in a given place. For each plot, we calculated both the unweighted indicator values, based upon the presence or absence of species, and weighted indicator values, taking into account the percent cover of species.

From the series of measurements of water table depth, we calculated the mean value and the range between the maximum and minimum water table depth as a measure of water conditions in that place. Using Spearman rank correlation, we determined the relationship between the Ellenberg’s soil moisture value F and the mean water table level for each sample plot (S2 Table)

Employing Kruskal–Wallis (one-way ANOVA on ranks**)** test we compared the distributions of Ellenberg indicator values for sample plots located in the undisturbed mire (MU) with these located in the disturbed mire (MD) and in the whole area of the reserve, containing open mires, partly overgrown mires and pine swamp forests. Using the same method, we compared the distributions of mean values of water table depth and range of water table changes for sample plots located in the Undisturbed mire (MU) in the disturbed mire (MD) and in the entire Kosowo bog reserve. Age distributions of tree growing in the disturbed mire and in the undisturbed mire were compared using the Kolmogorov-Smirnov two sample test.

For the purpose of investigation of the combined effect of different environmental factors on tree age and tree height we built a linear mixed model. In this model, we used independent quantitative factors expressed as weighted averages of the relevant Ellenberg’s indicator values as fixed effects. As comparing the forest versus open mire would yield very imprecise estimation of variance [38], we used the classification of plant communities into six different units; two types of open mire *Sphagnetum magellanici* (disturbed and undisturbed), two types of forest communities (*Vaccinio uliginosi-Pinetum* and *Vaccinio uliginosi-Betuletum*) and two type of transitional character, with disturbed/undisturbed *Sphagnetum magellanici* mires already invaded by pine trees. In our analyses we considered this variable as a random effect.

## Results

**Fig 1:**
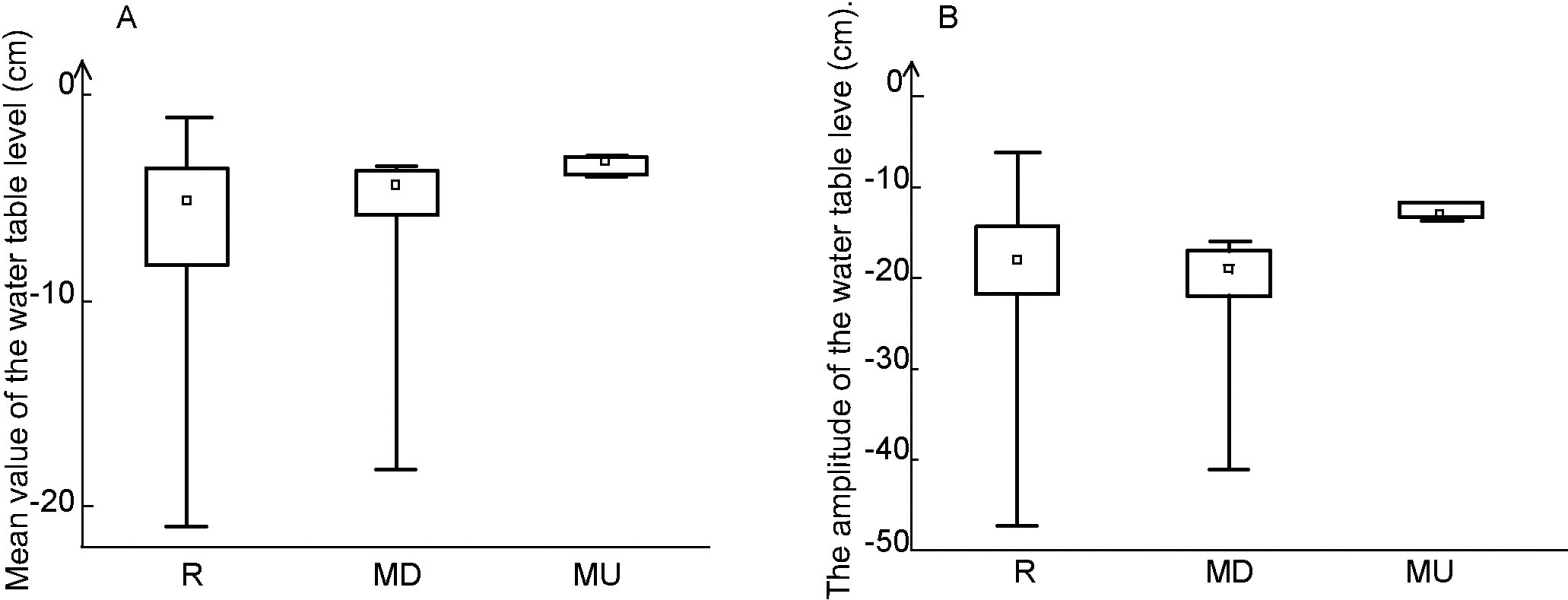
Comparison of mean values (A) and amplitudes (B) of the water table level in the entire reserve (left), in the disturbed mire (middle) and in the undisturbed mire (right).

Water table level ranged from 0 to -45 cm; the average value was closest to the ground surface in case of the undisturbed mire, and slightly deeper and much more variable in the disturbed mire and in the whole area of the reserve (Fig 1A). Similarly, the amplitude of water level was smallest in the undisturbed open mire (12 cm, with very low variability among the sample plots), and slightly bigger and more variable in the disturbed mire and in the reserve as a whole (Fig 1B). Since neither the water table height nor the variability in water table height turned out to be normally distributed, we used Levene’s test (which is known for being less sensitive to deviations from normality) to compare the variations between two mires. We obtained *p*-value level equal to 0.0011 in the first case and 0.018 in the second case. These results suggested statistically significant differences in the levels of variation between both mires.

**Fig 2:**
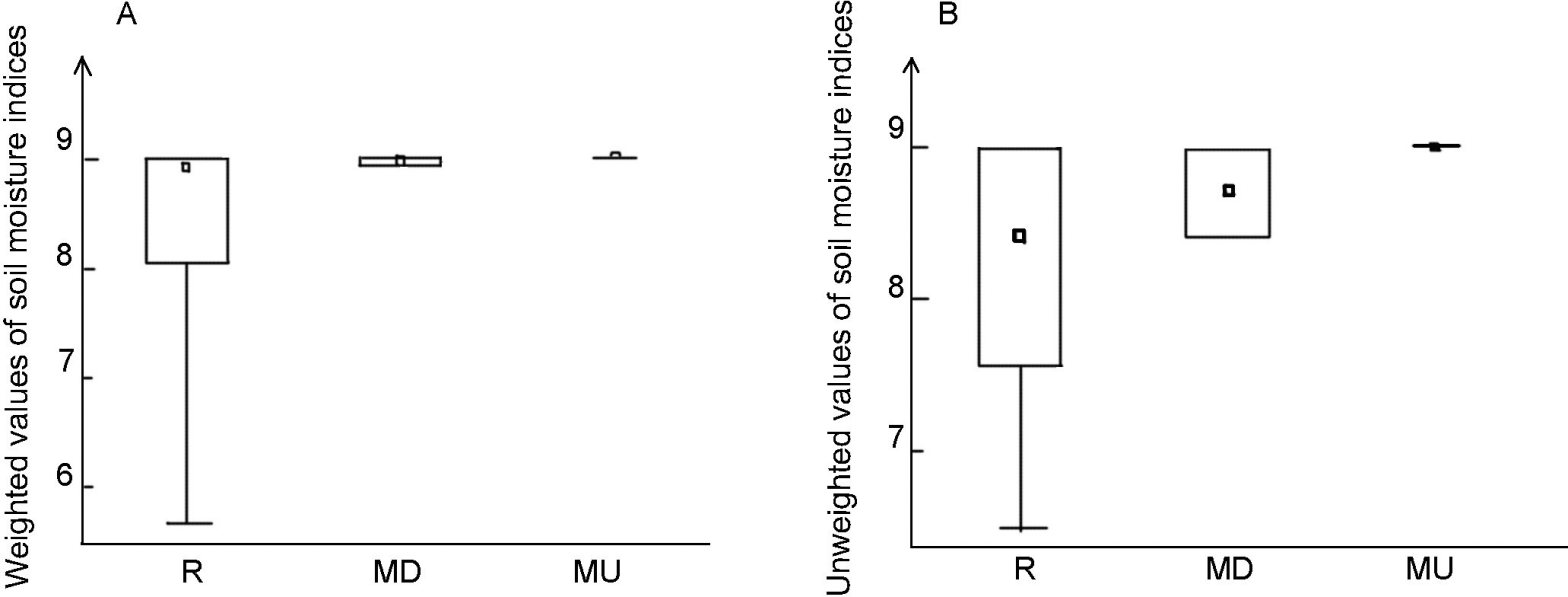
Comparison of unweighted (A) and weighted (B) values of Ellenberg’s soil moisture indices in the entire reserve (left), in the disturbed mire (middle) and in the undisturbed mire (right).

Calculating the unweighted Ellenberg indicator values for moisture (F) yielded consistently high values with little variation in the two open mires, while in the rest of the reserve there were some plots with moderate values of moisture indicators (Fig 2A). The highest values of moisture index and the lowest variation rate was found in the undisturbed mire. The weighted moisture indices were consistently high for undisturbed mire, slightly lower and much more variable in the disturbed mire, and much lower and even more variable for the entire reserve (Fig 2B). Using Levene’s test, we got confirmation of statistically significant differences in the level of variations in weighted moisture indices between both mires, with *p*-value less than 0.00001.

Analysis of the rank correlation between the average water table level and the Ellenberg’s indicator values for soil moisture (F) showed no significant correlation between these two variables (R = 0.0760 *p* = 0.6691). Both measures of local wetness indicate no significant differences between the disturbed and undisturbed mires. The direct measurements using piezometers had been conducted in one year, so they can be affected by a specific weather pattern of a given year. However, the estimates based upon the species composition of the plant cover (Ellenberg’s indicator values) are more stable, as the changes in mire vegetation are relatively slow.

Very similar results were obtained for the Nitrogen and soil reaction values; they were extremely low in open mires, and ranged from extremely low to moderately low throughout the reserve.

**Fig 3:**
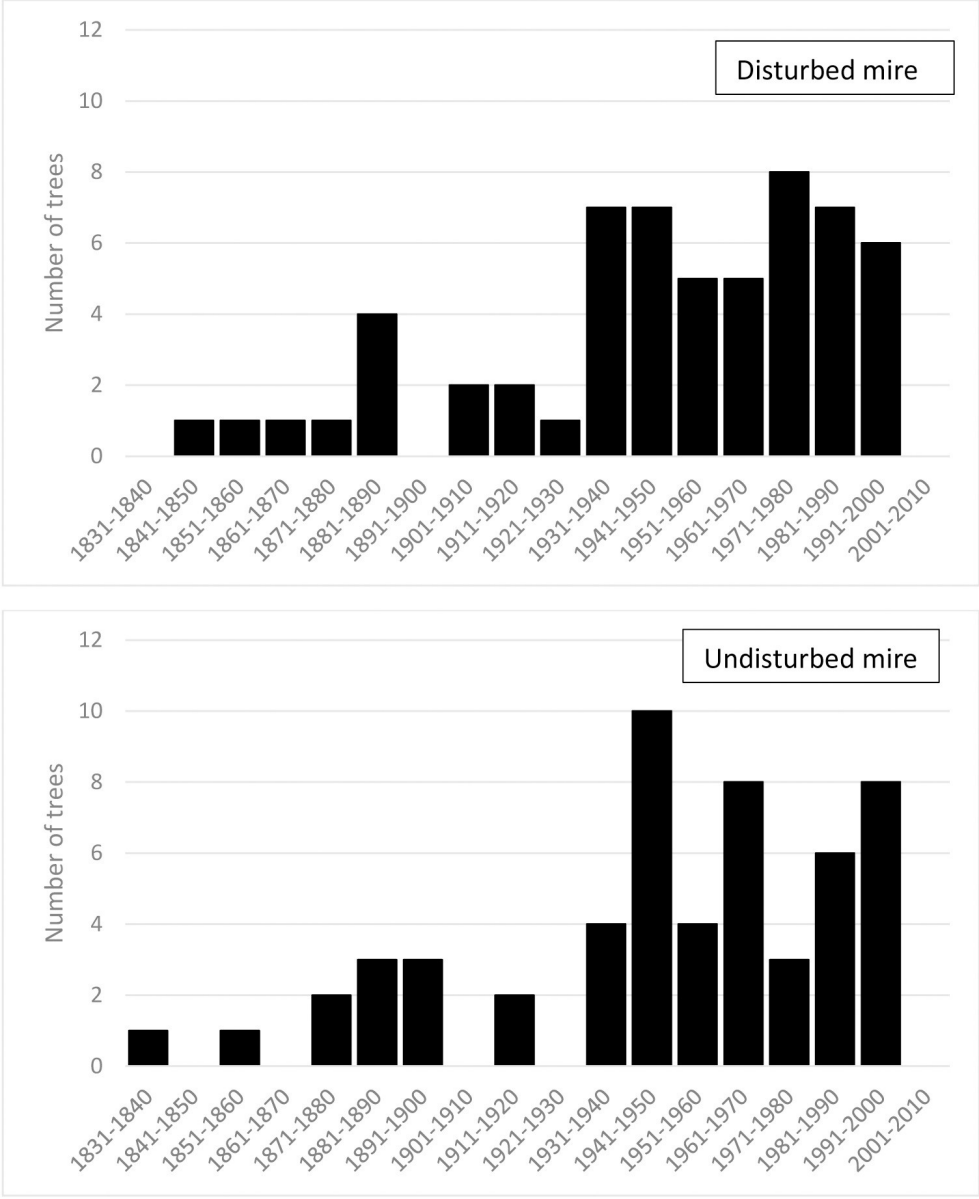
Number of the trees established in open mires of the northern part of Kusowo Bog Nature Reserve in consecutive decades during the period 1837–2011.

Trees were present along the whole transect on both mires. The oldest trees got established in the disturbed mires in 1844 and the youngest ones in 1985. In case of the undisturbed mire the respective years of the oldest and youngest tree establishment were 1837 and 1975. The number of the trees established in the consecutive decades of the analysed period is presented in the Fig 3.

Fig 3 shows clear phases of the tree establishment Comparison of tree encroachment of both investigated mires using Kolmogorov-Smirnov test showed no significant differences between disturbed and undisturbed mire (*p* >0.1), whilst trees on both mires displayed similar patterns in time of establishment. Up to 1860’s the rate of tree encroachment was low (1 tree/10 years, Fig 3), and then it increased until the end of 20th century (Fig 3).

**Fig 4:**
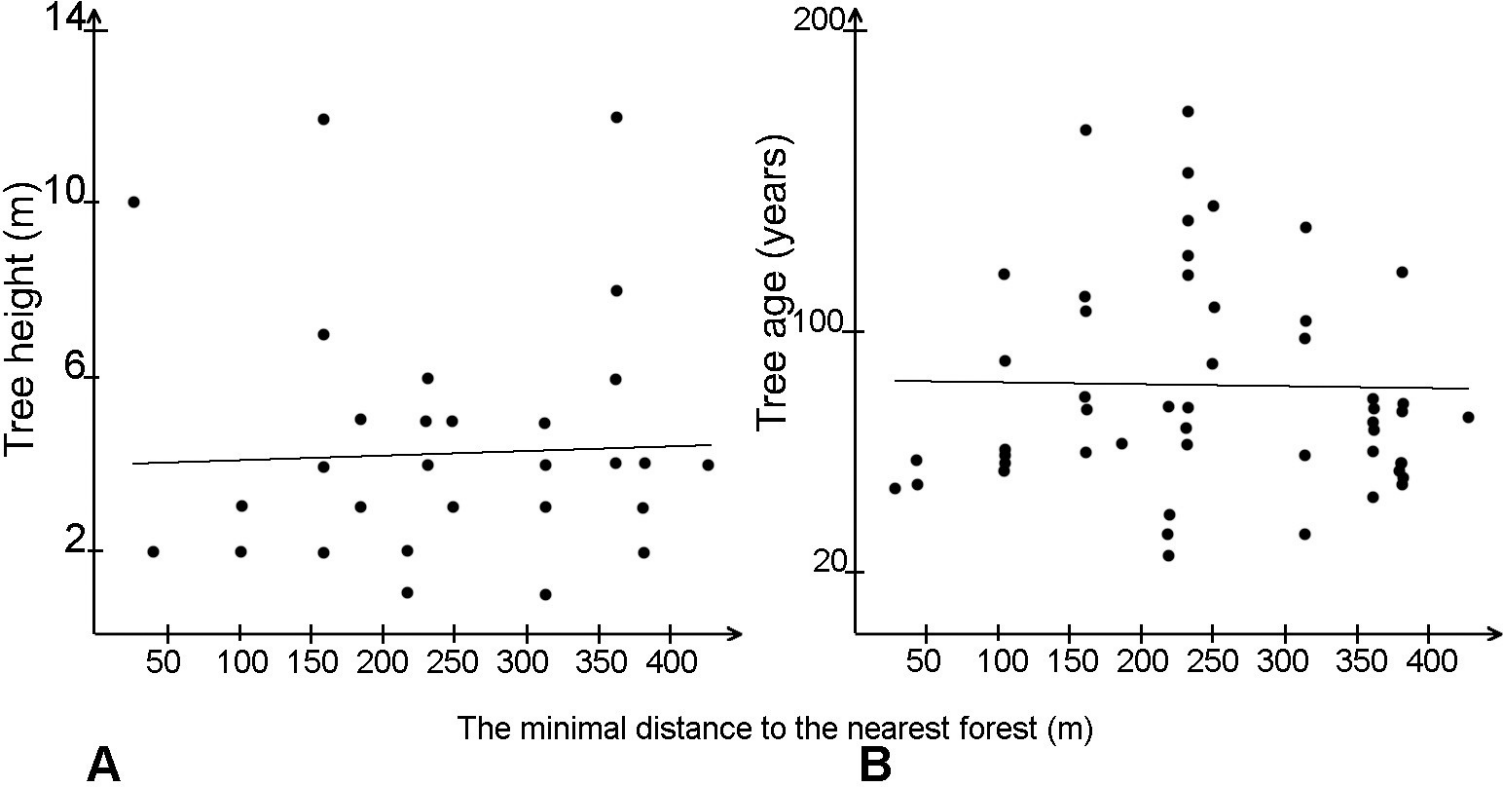
The relationship between the distance from the nearest forest edge and tree height (A) and tree age (B).

The pattern of tree encroachment disclosed by the tree age analysis shows differences between the directions of tree establishment during the investigated period, but without any clear relationship with the location of the open drainage ditches. The relationship between the distance from the edges of already existing forest and the age and height of trees established in open mires e was very weak (Fig 4).

**Fig 5:**
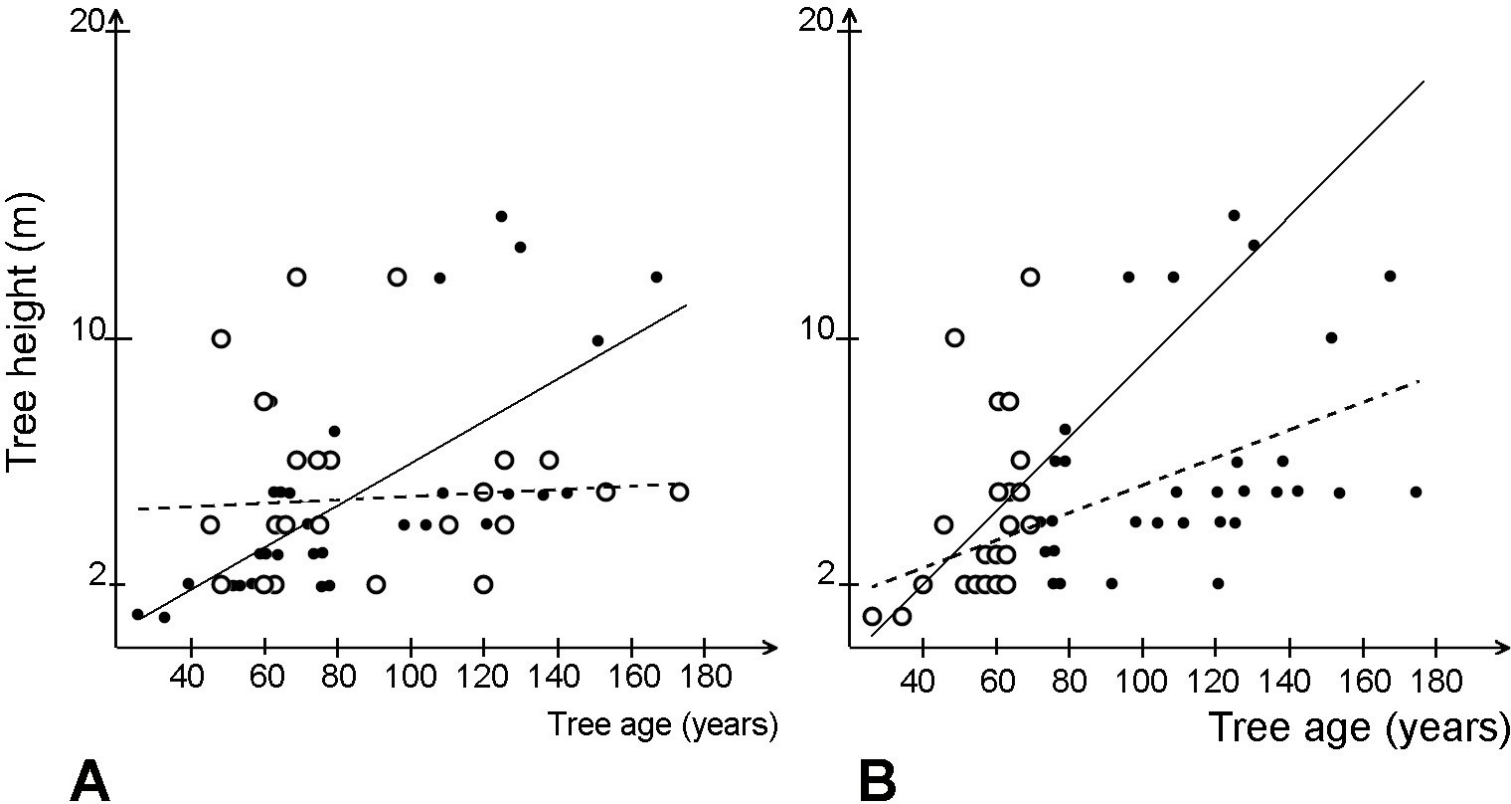
Relationship between tree age and tree height.

The relationship between tree age and tree height was also weak, especially in the undisturbed mire (Fig 5A). That was especially pronounced among the trees that were at least 70 year old. For trees younger than 70 years the relationship between age and height was slightly stronger (Fig 5B)

The analysis using linear mixed model concerned mainly the combined effect of soil reaction index and other environmental factors (water table depth and other Ellenberg’s indices) as fixed effect and the plant community type as random effect (Table 1). However, there was no statistical model in which soil reaction turned out to be statistically significant.

**Table 1:** Analysis of the linear model relating tree height and tree age to the environmental variables.

Variable\ *p*-value	Light	Temperature	Continentality	Moisture	Nitrogen
Tree height	<0.00001 *VB	**0.0008318**	**0.001093**	<0.000001 *VB	<0.00001 *VB
Tree age	0.008462 *SM2	0.005309 *SM2	0.007494 *SM2	0.008916 *SM2	0.008003 *SM2

Abbreviations: VB- *Vaccinio uliginosi-Betuletum*, SM2 –undisturbed mire *Sphagnetum magellanici*. Grey colour: only the plant community type was significantly affecting the outcome; Black colour: both the indicator values and plant community type were significant.

Investigation of the association between tree height and other environmental factors suggests significantly strong connection with soil moisture (with *p*-value < 0.00001) and soil nitrogen content (with *p*-value < 0.0000001) with significant difference of *Vaccinio uliginosi-Betuletum* plant community type in comparison to disturbed *Sphagnetum magellanici* as reference type of plant community, a variable which explains 55% of the variance of results between the outcomes. Similarly, the analysis of the relationship between tree age and other environmental factors yielded a significant difference of the undisturbed *Sphagnetum magellanici* vegetation SM2 type in comparison to the disturbed *Sphagnetum magellanici* SM1, explaining 26% of the total variance of the outcome (Table 2).

**Table 2:** Percent of variance of the dependent variable (that has left after it had been explained by fixed effects) explained by the differences between plant community types.

Variable\ % of explained variance	Light	Temperature	Continentality	Moisture	Nitrogen
Tree height	**50%**	**12%**	**16%**	**56%**	**50%**
Tree age	**25%**	**36%**	**31%**	**26%**	**26%**

## Discussion

The results of measurements of the water table and the calculated moisture indicator values showed that in the open mires the water level is still relatively high compared to other raised peat bogs in this part of Europe. The undisturbed mire showed slightly more stable moisture conditions compared to the open mire that had been drained in the past. We need to take into consideration that the year 2010 was the wettest year on the record for many localities in Poland [39], so the moisture conditions during this study were probably much better than before and after. Nevertheless, a single exceptional year could not change the floristic composition and the Ellenbergs’s indicator values derived from it. The results of linear model analysis showed that the plant community type and some of Ellenberg’s indicators affected the height and age of trees encroaching on the open mire. However, among many environmental variables only soil reaction was significant in all cases, while soil moisture was never a significant factor. Also the water table level measured with piezometers was never a significant factor in linear models for tree height and tree age in the open mires. The fact that comparison of Ellenberg’s indicator values for soil moisture did not yield significant differences between disturbed and undisturbed mires suggests that moisture conditions in both open mires were indeed similar.

According to our first hypothesis, there should be more trees established in the drained part of the Kusowo Peat bog. Our analyses showed, that forest succession onto the open mire occurs at the entire area of the bog, also in parts that had not been directly affected by drainage ditches. This result suggests that our first hypothesis was unsupported. Apparently some parts of the bog seem to be more susceptible to tree encroachment. Several earlier studies found, that the current tree establishment is more intense in the vicinity of already existing forest patches [16, 19, 32]. However, the analysis of the relationship between the metrics of tree encroachment (tree age, tree height) and the distance from the nearest forest edge showed no significant trend. Our second hypothesis was that the intensity of tree encroachment was greatest just after building the ditches and then it declined down. The more intensive tree encroachment of trees in the 1880s, as well as in the 1930s-1950s suggests some reaction to drainage operation, as the most probable establishment of drainage ditches took place in preceding periods, according to the historical and cartographic sources [33]. Peaks in tree encroachment in the 1860’s and the 1940’s can be directly related to the drainage operations being conducted at that time according to historical materials (map of the bog with the locations of the ditches from 1934). Similar processes of increasing the rate of forest succession onto open mires was documented from other locations [24, 28]. According to the study by Sarkkola [40], the number of trees encroaching onto open mires during the first 20 years after drainage operations can double or triple, which stays in line with the phase of intensive tree encroachment detected in Kusowo Bog. So our second hypothesis is partly confirmed by the results of this study.

Increase in the tree cover as the effect of the drainage has been reported in other studies regarding raised bogs in Poland [23]. In many cases the plausible explanation of intensive tree encroachment is the renewal of drainage system, as it happened in Stążki Baltic bog [23, 26] and Czarne Bagno raised bog [28]. However, records of management activities inspected in archive of Kusowo Bog Manager, available since early 1970’s confirm no drainage operations since that time. Now, as the whole area is protected as a nature reserve, no maintenance of the drainage ditches is allowed and we can expect that the remnants of the drainage ditches will be gradually filled with organic matter and eventually disappear.

Distinct phases in natural regeneration of Scots pine on raised bogs were reported by Brūmelis et al. [41]. Our study also showed an irregular pattern of tree appearance. Ågren and Zackrisson [14] related phases of tree encroachment on mires to the climatic conditions prevailing in bogs during particular periods of time. Overgrowing of open mires by trees indicates significant events as vegetation of open bogs is considered very stable in given climatic conditions [11, 12, 42, 43]. According to some studies, the gradual increase of annual temperatures is more important for the advance of tree succession than temporary droughts or exceptionally hot summers [44].

Tree age analysis showed that no trees got established after the year 2000. However, some young pines were found in the list of plants recorded while making the phytosociological relevés according to the Braun-Blanquet method [34]. The lack of young pines along transects may suggest greater mortality of the youngest trees currently germinating on the bog surface. The most plausible explanation for that could be the rise of water level and impoverishment of the tree growth condition in comparison to the previous years. Sarkkola [40] reported constant increase in mortality of trees during 50 years after cessation of drainage. Poor representation of the young generation of Scots pine on mires was reported from Białowieża Primeval Forest [45] where significant changes in vegetation pattern were recorded. That may indicate regional changes in vegetation following climate change and agrees with the results of this study.

The relationship between tree height and tree age was relatively weak, especially in older trees. Macdonald and Yin [46] reported close correlation between tree age and size only in the first 20–25 years. Socha [47] found that the younger the tree stand during the drainage the stronger is its increment reaction to the changes in water regime. Cedro and Sotek [33] who analysed the radial increment of Scots pine trees in the Kusowo Peat Bog recorded six periods of accelerated radial growth between 1840 and 2010. Four of those coincided with the periods of more numerous tree establishment found in this study. Especially the years 1946–1950, 1975–1980 and the period around the years 2000 were characterized by large radial increments and the establishment of numerous trees. In the same study [33], the role of climatic factors in shaping radial increment of Scots pines growing in Kusowo peat bog was also documented; the authors suggested, that the relatively fast radial increments of trees around the year 2000 could be explained by the accumulation of relatively warm and dry years in the period between 1990 and 2000.

Spatial pattern of forest succession on the Kusowo bog seems not to be related to the proximity of already existing stands. New trees were established both from adjacent stands located on the bog edges and in the central part of bog dome, which is relatively distant from the tree stands. A similar Lack of connection between the location of existing stands, presence of open drainage ditches and establishment of new trees was also reported by Langanke et al. [27]. This is understandable taking into account that Scots pine *Pinus sylvestris* is a species characterized by long-distance seed dispersal [41] and the distances to the nearest forest edge recorded in the Kusowo Bog are within the range of dispersal ability of that species. Recent expansion of trees and shrubs into the areas formerly covered by non-woody vegetation is now a large-scale phenomenon, recorded among the others in the vast areas of Siberia, and the proximity to the forest edge is not the only driver of that process [48].

Irregular pattern of tree encroachment suggests another factor linked to the drainage or following drainage operations that influence some patches of bog dome more than the others. The most likely factor differentiating tree encroachment at local scale is the microrelief of bog surface. A number of papers point to hummocks as the most favourable sites for tree growth [16, 31, 32]. However, during the drainage operations hummocks become less favourable microsites due to faster and stronger drying than the microsites located lower [46]. On the other hand, hummock structures can dwindle after drainage operations [25]. Because of that it is risky to assume that the present microrelief of the bog surface resembles the one before the drainage works had started and to explain the differences in tree encroachment by the current topographic factors.

## Conclusions

- Although the forest succession has been progressing over the last 180 years in the analysed peat bog, there is no clear differences in the rate of tree encroachment between the disturbed and undisturbed parts of the mire
- The more intense tree establishment in the decades following drainage operation suggest, that the draining operation probably enhanced forest succession onto open areas at the scale of the entire bog
- As there was no clear relationship between the water table level and the size or age of trees invading the open mires, the decrease in water table level is probably not the only factor influencing tree establishment and growth
- Differences in the rate of forest succession in various parts of the bog result mainly from differences in growth rates of trees, and not from differences in time of tree establishment

## Supporting information

S1 Table Characteristics of the trees established on the mires. (DOCX)

S2 Table Characteristics of the habitats and water conditions. (DOCX)
