# Supplementary material for: Drainage ditches enhance forest succession in a raised bog but do not affect the spatial pattern of tree encroachment
Source: PLoS One. 2021 Mar 18;16(3):e0247760. doi: 10.1371/journal.pone.0247760 (PMC7971578; doi:10.1371/journal.pone.0247760)
Supplement: S1 Table — (DOCX) [file pone.0247760.s001.docx]

S1 Table. Characteristics of the trees established on the mires.

| Distance along the transect line [m] | Tree height [m] | tree age [years |
| --- | --- | --- |
| 80 | 14 | 125 |
| 90 | 13 | 130 |
| 100 | 12 | 167 |
| 110 | 12 | 108 |
| 120 | 7 | 79 |
| 130 | 5 | 63 |
| 140 | 3 | 63 |
| 150 | 2 | 76 |
| 160 | 1 | 33 |
| 170 | 2 | 40 |
| 180 | 1 | 26 |
| 190 | 3 | 74 |
| 200 | 3 | 61 |
| 210 | 5 | 66 |
| 220 | 5 | 65 |
| 230 | 5 | 109 |
| 240 | 5 | 142 |
| 250 | 3 | 90 |
| 260 | 5 | 136 |
| 270 | 3 | 60 |
| 280 | 4 | 104 |
| 290 | 4 | 98 |
| 300 | 0 | 0 |
| 310 | 1 | 33 |
| 320 | 2 | 77 |
| 330 | 2 | 56 |
| 340 | 3 | 75 |
| 350 | 2 | 52 |
| 360 | 2 | 54 |
| 370 | 4 | 121 |
| 380 | 4 | 72 |
| 390 | 8 | 62 |
| 400 | 5 | 127 |
| 410 | 10 | 151 |
| 840 | 25 | 122 |
| 850 | 12 | 69 |
| 860 | 8 | 61 |
| 870 | 6 | 78 |
| 880 | 4 | 46 |
| 890 | 6 | 68 |
| 900 | 6 | 76 |
| 910 | 4 | 68 |
| 920 | 4 | 63 |
| 930 | 4 | 75 |
| 940 | 4 | 125 |
| 950 | 5 | 120 |
| 960 | 6 | 125 |
| 970 | 5 | 174 |
| 980 | 5 | 153 |
| 990 | 6 | 138 |
| 1000 | 4 | 111 |
| 1010 | 2 | 60 |
| 1020 | 2 | 76 |
| 1030 | 2 | 120 |
| 1050 | 2 | 91 |
| 1060 | 2 | 61 |
| 1070 | 2 | 55 |
| 1080 | 3 | 58 |
| 1090 | 2 | 58 |
| 1100 | 2 | 50 |
| 1110 | 10 | 49 |
| 1120 | 12 | 96 |
