# Supplementary material for: Drainage ditches enhance forest succession in a raised bog but do not affect the spatial pattern of tree encroachment
Source: PLoS One. 2021 Mar 18;16(3):e0247760. doi: 10.1371/journal.pone.0247760 (PMC7971578; doi:10.1371/journal.pone.0247760)
Supplement: S2 Table — (DOCX) [file pone.0247760.s002.docx]

S2 Table. Characteristics of the habitats and water conditions.

| Distance along the transect [m] | Sample plot number | Mean water table [cm] | Mean water table during the growing season [cm] | Lowest water table [cm] | Highest water table [cm] | Amplitude (cm) | Ellenberg's Indicator Value | | | | | |
| --- | --- | --- | --- | --- | --- | --- | --- | --- | --- | --- | --- | --- |
|  |  |  |  |  |  |  | Light availability | Temperature | Climatic continentality | Soil humidity or moisture | Reaction (soil or water acidity/pH) | Nitrogen (but really soil fertility or productivity, and not mineral nitrogen) |
| 132 | I.01 | -17.0 | -21.1 | -42.0 | -6.0 | -36.0 | 5.1 |  | 4.9 | 9.0 | 2.0 | 2.9 |
| 222 | I.03 | -2.3 | -3.2 | -8.9 | 0.0 | -8.9 | 7.1 | 4.5 | 3.7 | 8.9 | 1.9 | 1.0 |
| 346 | I.05 | -5.1 | -7.0 | -17.7 | 0.0 | -17.7 | 5.8 | 5.0 | 5.0 | 8.6 | 1.7 | 2.6 |
| 436 | I.07 | -4.6 | -7.3 | -18.8 | 0.0 | -18.8 | 5.6 | 5.0 | 4.7 | 8.4 | 1.8 | 2.6 |
| 518 | I.09 | -5.3 | -7.9 | -18.3 | 0.0 | -18.3 | 5.6 | 5.0 | 4.8 | 8.8 | 1.8 | 2.6 |
| 664 | I.12 | -1.1 | -1.7 | -6.1 | 0.0 | -6.1 | 7.1 | 4.5 | 3.5 | 8.9 | 1.9 | 1.0 |
| 736 | I.14 | -4.8 | -7.3 | -19.3 | 0.0 | -19.3 | 5.6 | 5.0 | 5.0 | 9.0 | 1.7 | 2.7 |
| 896 | I.16 | -3.3 | -5.1 | -15.0 | 0.0 | -15.0 | 6.9 | 4.8 | 4.5 | 8.9 | 1.9 | 1.2 |
| 1035 | I.18 | -1.7 | -2.7 | -11.0 | 0.0 | -11.0 | 7.1 | 4.5 | 3.3 | 9.0 | 1.9 | 1.0 |
| 1144 | I.20 | -16.9 | -20.8 | -34.8 | -6.5 | -28.3 | 5.2 |  | 5.0 | 7.3 | 1.9 | 2.8 |
| 125 | II.02 | -6.1 | -8.3 | -20.2 | 0.0 | -20.2 | 6.4 | 5.0 | 5.0 | 9.0 | 2.0 | 1.6 |
| 190 | II.04 | -3.6 | -5.6 | -16.0 | 0.0 | -16.0 | 7.1 | 4.4 | 3.2 | 9.0 | 1.9 | 1.0 |
| 255 | II.05 | -3.5 | -5.5 | -17.1 | 0.0 | -17.1 | 7.0 | 4.8 | 3.5 | 8.9 | 2.0 | 1.0 |
| 320 | II.06 | -5.8 | -8.9 | -22.0 | 0.0 | -22.0 | 7.1 | 4.3 | 3.3 | 9.0 | 1.9 | 1.0 |
| 380 | II.07 | -18.2 | -23.5 | -41.1 | 0.0 | -41.1 | 7.2 | 4.3 | 3.3 | 8.9 | 1.9 | 1.0 |
| 439 | II.09 | -8.3 | -12.4 | -31.8 | 0.0 | -31.8 | 6.7 | 4.0 | 4.4 | 8.9 | 1.8 | 1.5 |
| 511 | II.11 | -9.4 | -11.4 | -26.0 | -1.9 | -24.1 | 7.2 | 4.3 | 3.3 | 8.9 | 1.9 | 1.0 |
| 544 | II.12 | -5.2 | -7.9 | -18.2 | 0.0 | -18.2 | 7.0 | 5.0 | 3.8 | 8.9 | 1.9 | 1.1 |
| 601 | II.14 | -3.9 | -6.2 | -15.7 | 0.0 | -15.7 | 6.6 | 5.0 | 4.4 | 9.0 | 1.9 | 1.4 |
| 714 | II.20 | -3.8 | -5.9 | -17.5 | 0.0 | -17.5 | 5.1 | 5.0 | 5.1 | 7.3 | 2.0 | 2.8 |
| 820 | II.21 | -3.2 | -4.9 | -14.3 | 0.0 | -14.3 | 5.3 | 5.0 | 5.0 | 5.7 | 1.8 | 2.9 |
| 906 | II.23 | -3.2 | -4.7 | -11.7 | 0.0 | -11.7 | 7.0 | 4.8 | 4.3 | 9.0 | 1.9 | 1.1 |
| 1033 | II.25 | -3.9 | -5.1 | -13.1 | 0.0 | -13.1 | 7.3 | 4.3 | 3.2 | 9.0 | 1.7 | 1.0 |
| 1105 | II.27 | -11.2 | -12.6 | -27.4 | -2.0 | -25.4 | 5.3 | 5.0 | 5.0 | 6.1 | 1.8 | 2.8 |
| 21 | III.01 | -4.4 | -5.9 | -17.1 | 0.0 | -17.1 | 5.3 | 5.0 | 4.9 | 7.7 | 2.0 | 2.8 |
| 71 | III.03 | -3.2 | -5.0 | -13.5 | 0.0 | -13.5 | 7.5 | 4.6 | 3.1 | 9.0 | 1.5 | 1.0 |
| 150 | III.05 | -3.5 | -5.5 | -17.1 | 0.0 | -17.1 | 5.2 | 5.0 | 5.0 | 7.6 | 2.0 | 2.8 |
| 256 | III.06 | -5.2 | -8.4 | -18.8 | 0.0 | -18.8 | 7.3 | 5.0 | 3.1 | 9.0 | 1.7 | 1.0 |
| 363 | III.08 | -15.9 | -20.9 | -45.0 | 0.0 | -45.0 | 5.3 | 5.0 | 4.8 | 7.0 | 1.9 | 2.9 |
| 445 | III.13 | -6.8 | -7.6 | -14.2 | 0.0 | -14.2 | 6.7 | 4.7 | 4.9 | 9.0 | 1.9 | 1.5 |
| 495 | III.15 | -20.9 | -25.7 | -44.1 | 0.0 | -44.1 | 5.2 | 5.0 | 5.0 | 9.0 | 2.0 | 2.8 |
| 543 | III.19 | -13.4 | -19.0 | -41.6 | 0.0 | -41.6 | 7.0 | 4.7 | 3.9 | 8.8 | 1.9 | 1.1 |
| 593 | III.21 | -3.9 | -5.9 | -16.2 | 0.0 | -16.2 | 7.2 | 4.5 | 3.0 | 8.9 | 1.8 | 1.0 |
